# Epitopes of MUC1 Tandem Repeats in Cancer as Revealed by Antibody Crystallography: Toward Glycopeptide Signature-Guided Therapy

**DOI:** 10.3390/molecules23061326

**Published:** 2018-05-31

**Authors:** Dapeng Zhou, Lan Xu, Wei Huang, Torsten Tonn

**Affiliations:** 1 Shanghai Pulmonary Hospital Affiliated with Tongji University School of Medicine, Shanghai 200092, China; 2 Laboratory of Antibody Structure, Shanghai Institute for Advanced Immunochemical Studies, ShanghaiTech University, Shanghai 201203, China; 3 CAS Key Laboratory of Receptor Research, Shanghai Institute of Materia Medica, Chinese Academy of Sciences and iHuman Institute, ShanghaiTech University, Shanghai 201203, China; huangwei@simm.ac.cn; 4 Institute for Transfusion Medicine Dresden, German Red Cross Blood Donation Service North-East, D-01307 Dresden, Germany; t.tonn@blutspende.de; 5 Medical Faculty, Carl Gustav Carus Technical University Dresden, D-01307 Dresden, Germany

**Keywords:** MUC1, tandem repeat, antibodies, glycopeptide, co-crystal

## Abstract

Abnormally *O*-glycosylated MUC1 tandem repeat glycopeptide epitopes expressed by multiple types of cancer have long been attractive targets for therapy in the race against genetic mutations of tumor cells. Glycopeptide signature-guided therapy might be a more promising avenue than mutation signature-guided therapy. Three *O*-glycosylated peptide motifs, PDTR, GSTA, and GVTS, exist in a tandem repeat HGVTSAPDTRPAPGSTAPPA, containing five *O*-glycosylation sites. The exact peptide and sugar residues involved in antibody binding are poorly defined. Co-crystal structures of glycopeptides and respective monoclonal antibodies are very few. Here we review 3 groups of monoclonal antibodies: antibodies which only bind to peptide portion, antibodies which only bind to sugar portion, and antibodies which bind to both peptide and sugar portions. The antigenicity of peptide and sugar portions of glyco-MUC1 tandem repeat were analyzed according to available biochemical and structural data, especially the GSTA and GVTS motifs independent from the most studied PDTR. Tn is focused as a peptide-modifying residue in vaccine design, to induce glycopeptide-binding antibodies with cross reactivity to Tn-related tumor glycans, but not glycans of healthy cells. The unique requirement for the designs of antibody in antibody-drug conjugate, bi-specific antibodies, and chimeric antigen receptors are also discussed.

## 1. Introduction

Cancer glycoproteins have been targets for diagnosis and therapy since President Nixon signed the National Cancer Act of 1971 [1]. Decreases in glycosylation occurred in membrane glycoproteins represent a most common form of tumor-specific alteration in *O*-linked glycosylation [2]. The truncation or absence of the glycosylation expose novel antigens or cryptic epitopes, such as Tn epitope, an abnormally exposed *O*-linked GalNAc residue. Tn epitope was discovered as a carbohydrate antigen on the surface of red blood cells from patients with a very rare Tn-syndrome [3,4], an acquired hemolytic anemia with polyagglutinability of red blood cells due to a new factor present in normal human serum (Anti-Tn). Thurnher et al. found that the suppression of the core-1 β3-galactosyltransferase (EC 2.4.1.122) enzyme activity caused the exposure of Tn residue in Tn syndrome [4]. It was later found that the enzyme complex is composed of the core-1 β3-galactosyltransferase and Cosmc chaperon protein. The molecular mechanism of Tn syndrome was finally characterized as epigenetic silencing of Cosmc gene through hyper-methylation of its promoter region [5]. Tn antigen was soon found to be expressed by majority (90%) of cancer cells [6,7,8,9,10]. Several other abnormally expressed sugar epitopes structurally related to Tn were also discovered, including STn (sialyl Tn, [11,12]), T (Gal β3 GalNAc, [13,14,15,16,17]), ST (Neu5Ac α6 Gal β3 GalNAc, [18,19,20,21,22,23,24,25,26]), and others.

**Figure 1:**
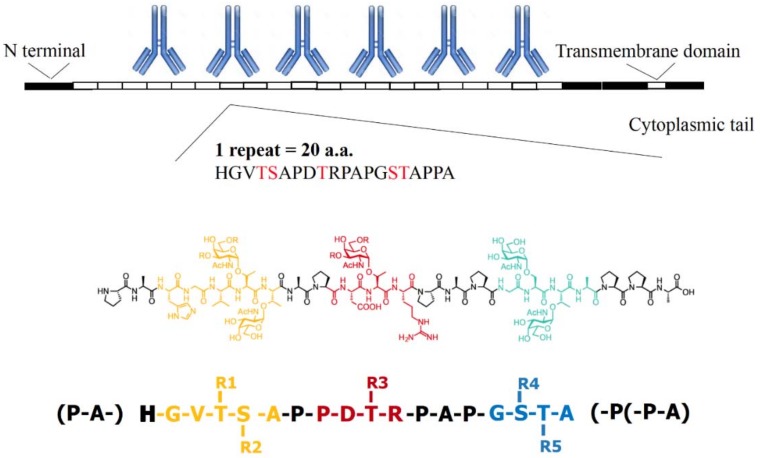
Peptide motifs (colored amino acids) and *O*-glycosylation sites (R) of the MUC1 tandem repeat sequence. MUC1, transmembrane glycoprotein, has variable number tandem repeat (VNTR) motif, which is composed of 20 amino acids that are heavily *O*-glycosylated at the serine and threonine residues. GVTS, PDTR, and GSTA are three glycosylated regions, with five *O*-glycosylation sites. In cancer cells, abnormally glycosylated MUC1 TR repeats are targets for binding by IgG molecules.

MUC1 is the most abundant protein in cancer cells which bear the abnormally glycosylated *O*-glycans [27]. The extracellular portion of mucins contains about 20 to 120 or more repeats of 20-22-residue sequence (HGVTSAPDTRPAPGSTAPPA) with five potential *O*-linked glycosylation sites through *N*-acetylgalactosamine to serine and/or threonine residues (Figure 1). The heavily glycosylated sequence of MUC1 is termed as tandem repeat due to its repeated occurrence. Tandem repeat sequence is the most abundantly *O*-glycosylated region of MUC1. MUC1 is normally expressed at low levels at the apical surface of most glandular epithelial cells, with normal pattern of *O*-glycosylation. In tumor settings MUC1 loses its polarity and normal pattern of glycosylation to expose GalNAc (Tn) residue. MUC1 has been ranked No. 2 of all 75 tumor-associated antigens as cancer vaccine targets evaluated by National Cancer Institute Translational Research Working Group, based on certain criteria, such as therapeutic function, immunogenicity, cancer cell specificity etc. [28].

### 1.1. The Sugar and Peptide Portions of MUC1 in Clinical Medicine

Previous clinical trials for MUC1-based cancer vaccines have been based on separated sugar and peptide portions. This may be caused by two factors. First, there were no knowledge about what glycopeptides are expressed by tumor cells. The obvious large structural diversity also begs the question whether personalized glycopeptide-epitopes should be targeted, or more “generic type” of glycopeptide-epitopes should be targeted. The clinical evidence to support the notion on using uniquely-mutated neoantigens as vaccine sources is still very limited [29]. Secondly, the chemical synthesis of glycopeptide was impractical in early era of research and development, although this is currently not an issue with the advent of Fmoc solid-phase peptide synthesis, better protection groups and development of chemoenzymatic process for the attachment of sugar moiety.

The abnormally glycosylated MUC1 in mucosal inflammation and in adenocarcinomas induces humoral immune responses to the mucin. Antibody targeting MUC1 may block MUC1-induced expression of genes associated with tumor invasion, angiogenesis and metastases and restore immune surveillance suppressed by abnormally overexpression of MUC1 on cancer cells. The MUC1-derived peptide sequences, including RPAPGS, PPAHGVT, and PDTRP, especially PDTRP motif, are the most frequent minimal epitopes being identified [30]. It has been suggested that with GalNAc (Tn-antigen) attachment, the peptide epitopes’ immunogenicity was greatly enhanced [31], presumably due to the carbohydrate-induced favorable conformational changes of the peptide backbone [32]. Therefore, the combination of peptide and sugar portions of a MUC1 glycopeptide is essential for vaccine design and antibody development. Several candidate vaccines and antibodies have been published, such as the tripartite glycopeptide vaccine composed of the immunoadjuvant Pam(3)CysSK(4), a peptide T(helper) epitope and an aberrantly glycosylated MUC1 peptide [33]. Immunization with this tripartite version of vaccine exhibited superior anti-tumor effect. More recently, a clinical trial with the MUC1 100 mer peptide plus Hiltonol adjuvant enlisted a panel of new human anti-MUC1 antibodies that recognized the common PDTR minimal epitope with high affinity and exquisite in vitro anti-tumor effect and in vivo safety in human donors were reported [34]. The monoclonal antibody 5E5 [35] was generated using fully *O*-glycosylated (five sites per repeat) MUC1 glycopeptide conjugated to KLH as vaccine adjuvant. The antibody showed high cancer cell specificity and its scFv was used to construct a chimeric antigen receptors (CARs). The CAR-T cell did not recognize normal human cells and demonstrated potent antitumor effects [36]. The success of utilizing anti-MUC1 antibody in the CAR-T cell engineering strongly support targeting aberrantly glycosylated glycopeptide as a promising therapeutic approach. A high affinity antibody 16A was produced by immunizing mice with MUC1-transfected mutant cancer cells and showed significant glycopeptide binding preference and recognized RPAPGS(GalNac)TAPPAHG epitope [37].

While aberrantly glycosylated MUC1 is a viable anticancer immunotherapy target, the knowledge regarding the rule that govern the immunogenicity of certain MUC1 glycopeptide are still scarce. Crystallography along with computational approach will decipher the antibody recognition of glycan epitope and provide structural insights on epitope selection for vaccine design. A handful complexed structures of carbohydrate-binding antibodies with their cognate epitopes have been reviewed and provided an excellent overview on the antibody recognition mechanism of carbohydrate-based epitopes [38]. The “anchoring glycan motif” hypothesis was proposed as a common mechanism on how these carbohydrate-targeting antibodies modify cancer cell membrane and initiate antibody-dependent cytotoxity (ADCC) and complement dependent cytotoxicity (CDC) anticancer effect [39]. Nevertheless, structural overview on anti-MUC1 antibodies and antibody targeting tumor-associated antigenic glycopeptides in general have not been mentioned. This review will mainly focus on the molecular and structural basis responsible for the antibody binding to MUC1 tandem repeats and its potential as vaccine design and therapeutic antibody development.

Tn-antigen is abundantly expressed on the surface of various cancer cells, therefore, is among most studied carbohydrate antigens. Nevertheless, weak immunogenicity is associated with Tn carbohydrates due to the expression of the Tn-antigen on embryonic [40] and nonmalignant adult tissues which appear mostly restricted to upper digestive tract [41]. Structural elucidation of the clinically-relevant Tn-antigen in complexed with their cognate antibodies will be important to shed light on the minimal carbohydrate-based epitope identification and how to improve the immunogenicity and tumor-specificity in vaccine design and therapeutic antibodies discovery. Better knowledge and in-depth understanding of such complexed structures are needed, with only limited number of determined Tn-glycopeptide and antibody complexed structures available today.

## 2. Published Co-Crystal Structures: 3 Hypothesized Models of CDR (Complementarity Determining Region) Binding to Glycopeptide Signature

There are very few antibodies that have been co-crystalized with glycopeptide ligands. Three hypothesized models can be proposed based on current available data.

### 2.1. CDRs Bind to Sugar and Hypermutated FRs Bind to Peptide

**Figure 2:**
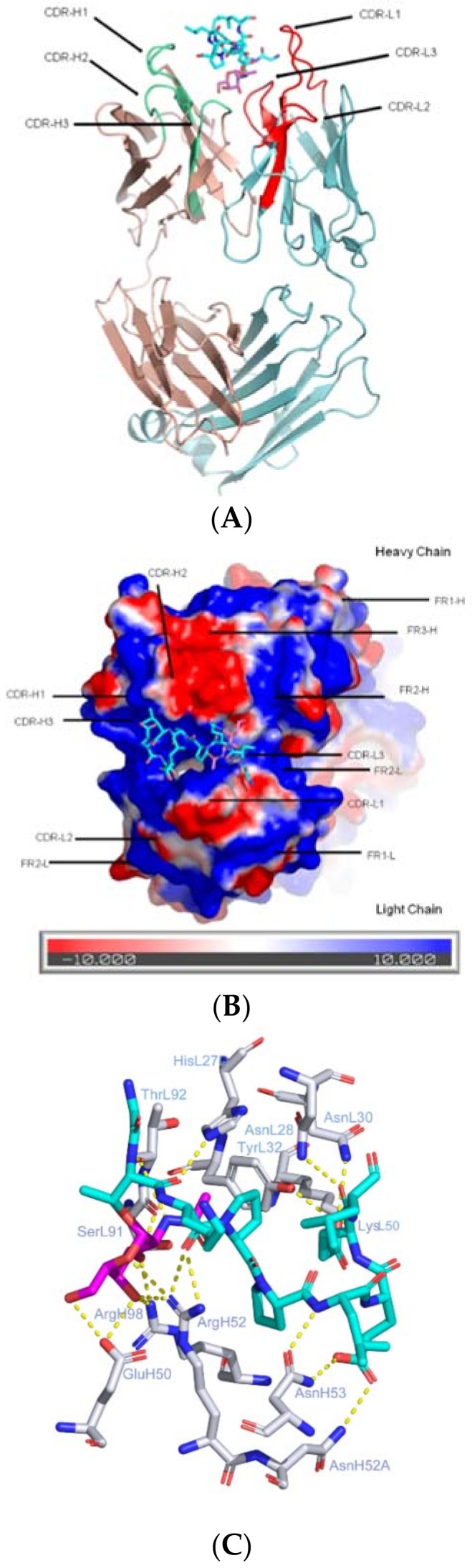
The CDRs of 237 mAb in complexed with tumor-specific glycopeptide of podoplanin. (**A**) The structure of 237 mAb in complex with glycopeptide (ERGT(GalNAc)KPPLEELS) (PDB ID: 3iet): The carbon atoms of glycopeptide are colored in cyan, with the sugar moiety GalNAc highlighted in hot pink (PDB ID: 5t78). The light chain is colored in light blue with CDR loops highlighted in red and labeled. The heavy chain is colored in pink with CDR loops highlighted in lime green. The carbohydrate is wedged in the binding pocket formed by CDRs L3, H1, H2 and H3; (**B**) Electrostatic surface potentials are colored red and blue for negative and positive charges, respectively, and white color represents neutral residues. The orientation of the antibody is adjusted to show the binding of the glycopeptide with more clarity; (**C**) Key interactions of the glycopeptide with the 237 mAb combining site. Hydrogen bond are depicted in yellow dotted lines. The sidechains of 237 mAb were depicted in white ball-and-sticks with the glycopeptide colored in blue. The GalNAc sugar moiety was highlighted in magenta. The residues on CDR heavy chains are labeled with H, those on CDR light chains are labeled with L.

This model fits the “anchored glycan motif” hypothesis that the sugar portion serves as the anchor to bind CDRs of an antibody [39]. One example of this type of antibody is 237 mAb [42,43], which binds to the glycopeptide ERGT(Tn)KPPLEELS derived from podoplanin [44]. Even though it does not belong to the anti-MUC1 antibody family, it was worth mentioning as the first anti-Tn-glycopeptide antibody complexed structure determined. The glycopeptide epitope is present within the extracellular domain of the glycoprotein podoplanin expressed by an aggressive fibrosarcoma spontaneously arising in an aging mouse [44]. A tumor-specific mutation in the chaperone gene Cosmc abolished the core 1 β3-galactosyltransferase enzyme activity. The disruption of *O*-glycan core 1 synthesis resulted in a single Thr *O*-linked GalNAc (i.e., a Tn antigen) attaching to peptide moiety of the epitope [43]. 237 mAb exhibited an elegant specificity toward glycosylated podoplanin, with absolutely no cross reactivity to the normal non-glycosylated podoplanin. The complexed structure of mAb237 and the glycopeptide helps to elucidate the striking glycan specificity of the antibody. The sugar part of glycopeptide is buried deeply in a “pocket” formed by the CDR regions of germline genes, while peptide portion of the antigen binds to the CDR variable loop regions (Figure 2). One structural feature worth mentioning is the sugar moiety forms strong hydrogen binding with a framework residue Glu-H50, which seems to confer the elegant specificity of 237 mAb to the glycopeptide.

The 237 mAb has undergone extensive somatic hypermutations, with 11 amino acid substitutions located in the heavy chain, 5 substitutions in the light chain. Most strikingly, 15 of the 16 mutations occurred within the framework regions, with only a single substitution occurring in the CDR of heavy chain (hA56 → hE56). It has been suggested that through extensive FR mutations from the germline sequences, mAb237 achieved its striking recognition specificity toward the glycopeptide through FR residues, albeit no hypermutations from the germline sequence were observed. FR mutations play essential roles in shaping the CDR loops and enable strong potency selection and broad neutralizing power in HIV vaccine design [45]. It is likely that the FR mutations in mAb237 provide similar functions. 

In addition, mAb237′s recognition specificity also includes distinguishing between disaccharides and monosaccharides, which is based on the shape and size of the binding pocket, where only a monosaccharide can be accommodated. In other word, the sugar moiety of mAb237′s epitope dominates the specificity. An unusual lower binding affinity (K_d_ = 0.14 μM) is associated with the glycopeptide binding to mAb237, comparing to traditionally observed for antibodies specific for peptides. The fact that the binding of the free sugar GalNAc or peptide to mAb237 could not be detected by SPR indicated the recognition specificity is achieved through the coordinated effect of both GalNAc and the peptide. The sugar portion of glycopeptide is deeply enveloped by a “pocket” formed by the CDR regions of germ-line genes (IGHV6-6*01 and IGKV1-110*01), while peptide portion binds to FR regions of mutated VH and VL (Figure 2).

The non-mutated germ-line IGHV6-6*01 and IGKV1-110*01 sequences that contain CDR regions of 237 may be related to Tn antigen binding. Other groups reported the binding to Tn antigen by non-mutated germ-line sequences from different IGHV and IGLV germ-line sequences [46]. For example, Yuasa et al. reported that MLS128 mAb, which binds to Tn sugar independently of peptide backbone, used IgHV1-78 germ-line VH sequence. 83D4 mAb also used IgHV1-78 germ-line VH sequence [47]. In contrast, mAbs MLS128 and 83D4, derived from IgHV1-78, can bind to GalNAc residues in ELISA assays, independent of peptide backbone. However, 237 mAb does not show detectable binding to GalNAc sugar structure alone by ELISA method, although the complex structure of GalNAc free sugar and Fab of mAb 237 could be obtained.

### 2.2. CDRs Bind to Both Sugar and Peptide Portions

There are very few anti-MUC1 antibodies either alone or in complexed with their cognate glycopeptide epitopes, even though a handful of anti-MUC1 antibodies have been developed [48]. SM3 was the first anti-MUC1 antibody to be crystallized with its cognate peptide repeat [49]. SM3 was raised against mucin stripped most of its carbohydrate [50]. It exhibited high selectivity against carcinoma-associated mucin in more than 90% breast cancers [51]. It recognizes the core repeating motif (Pro-Asp-Thr-Arg-Pro) [50] within the “knob-like” domain determined by 3D-NMR spectra of the MUC1 peptide [52]. The bound peptide adopted mainly extended conformation with no significant secondary structure and bind to the elongated binding groove that are encompassed by all six CDR loops. The large conformational change between the solution unbound structure and the antibody-bound peptide highlights the importance of the epitope conformation in the vaccine design.

**Figure 3:**
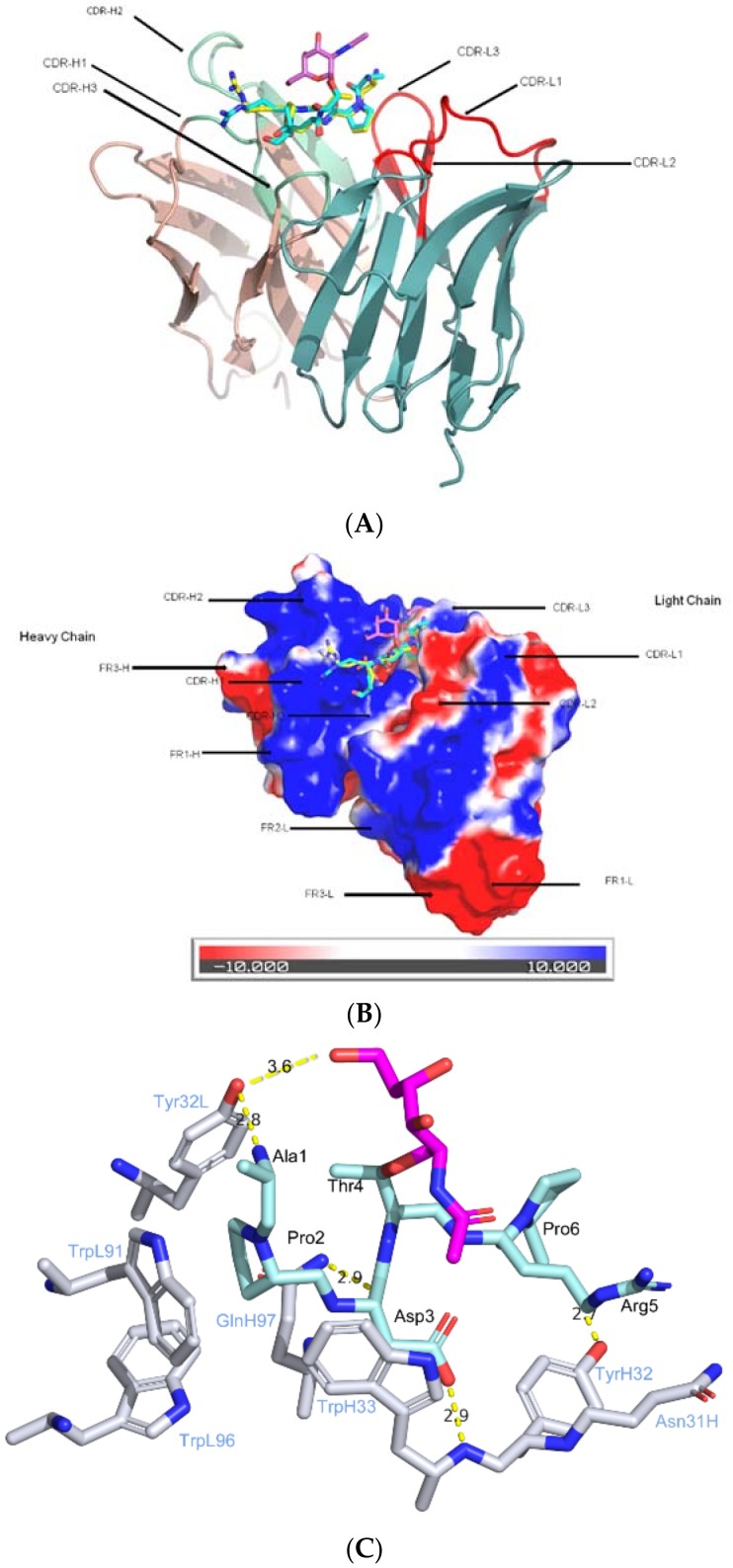
SM3 in complexed with MUC1 peptide and glycopeptide. (**A**) SM3 in complexed with MUC1 glycopeptide (SAPDTnRPAP) (PDB ID: 5a2i) (with carbon atoms in cyan and the sugar moiety highlighted in hot pink). The color codes for VL chains and CDR loops L1-3, as well as VH chains and CDR loops H1-3 are the same as in Figure 2; (**B**) Surface representation of SM3 with bound peptide and glycopeptide in the binding pocket. From the surface representation, the sugar moiety does not form extensive interactions with SM3, instead, similar to AR20.5 complexed structures, pointing towards the solvent; (**C**) Key residues involving in SM3 mAb-glycopeptide interactions. The color and label schemes are similar to Figure 2.

In 2015, Martinez-Saez et al. published the first co-crystal structure of SM3 in complexed with the tumor-specific glycopeptide APDT(Tn)RP [53] (Figure 3). The structure provided the molecular basis on how the glycans on the glycopeptide induced bioactive conformation for optimal antibody recognition as suggested by previous studies [54,55,56,57]. The binding affinities of the 20 amino-acid tandem repeat (AHGVTSAPDTRPAPGSTAPP) peptide and glycopeptide at Thr10 were determined by Bio-layer interferometry (BLI) There is a 3-fold difference between the K_d_ constants for the peptide (1.4 ± 0.13 μM) and glycopeptide (0.45 ± 0.072 μM), indicating epitope recognition differentiated by SM3. The peptide and glycopeptide bind to SM3 combining site in almost identical conformation, similar to that observed for SM3 in complexed with the “naked” peptide determined previously [49]. Surprisingly, while the peptide forms extensive hydrophobic and hydrophilic interactions with the combining site of the antibodies (Figure 3C), the GalNAc sugar is pointing towards the solvent and adopts a conformation that enables a relatively weak hydrogen bond between the hydroxymethyl group of GalNAc and the hydroxyl group of Tyr32L (~3.6 Å). The binding of the glycopeptide is further enhanced by the stacking of aromatic ring of Trp33H and *N*-acetyl group of the sugar. The extra interactions between the antibody and GalNAc seems to contribute partly to the recognition specificity of the glycopeptide. The GalNAc sugar on the glycopeptide also induces a preferred conformation that is suitable for antibody recognition, which seems to confer additional specificity.

### 2.3. CDRs Bind to Peptide Portion, while the Sugar Portion May Alter Peptide Confirmation

**Figure 4:**
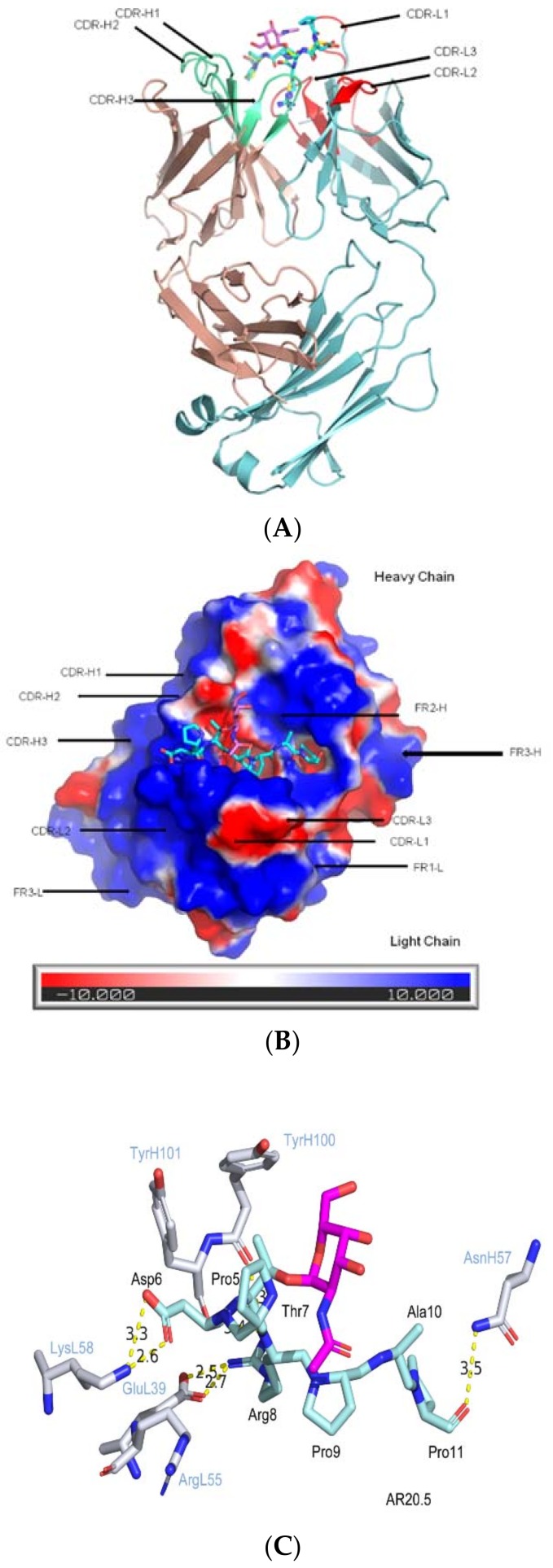
The CDRs of mAb AR20.5 bind to the peptide motif of MUC1 without direct contacts with sugar. (**A**) AR20.5 in complexed with glycopeptide (APDTnRPAP): The carbon atoms of glycopeptide are colored in cyan, with the sugar moiety GalNAc highlighted in hot pink (PDB ID: 5t78). The light chain is colored in light blue with CDR loops highlighted in red and labeled. The heavy chain is colored in pink with CDR loops highlighted in lime green; (**B**) Electrostatic surface potentials are colored red and blue for negative and positive charges, respectively, and white color represents neutral residues. The orientation of the antibody is adjusted to show the binding of the glycopeptide with more clarity; (**C**) Interactions of MUC1 glycopeptide with AR20.5 mAb binding pocket. The color and label schemes are similar to Figure 2.

In 2016, Movahedin et al. published the co-crystal structure of AR20.5 bound with APDT(Tn)RPAP [58] (Figure 4). AR20.5 (OncoQuest Inc., Edmonton, AB, Canada), is a murine anti-MUC1 monoclonal antibody (IgG_1_) generated with MUC1 from an ovarian cancer patient [59]. The 6-amino acid epitope (DTRPAP) with a single glycosylation at Thr within the tandem repeat represents the minimal epitope AR20.5 recognizes. An 8-mer synthetic peptide (APDTRPAP, K_d_ = 880 ± 123 nM) and its corresponding glycopeptide (K_d_ = 43 ± 3.6 nM) were used in a microscale thermophoresis (MST) for a binding analysis to AR20.5. Strikingly, the single GalNAc sugar moiety enhances the binding to the glycopeptide by 20-fold. From the complexed structures of 8-mer peptide and glycopeptide with AR20.5, the binding modes of the peptide portion are almost superimposable locating in a deep groove composed of the CDR loops L1, L2, H2 and H3. Similar to SM3, the sugar moiety binds in the surface groove without specific hydrophilic and hydrophobic interactions with the antibody. Only a long-distant hydrogen bonding and plausible aromatic ring stacking between the sugar and the antibody were observed. It is suggested the strong preference to glycopeptide epitope recognition is due to the presence of the sugar, which shifts the equilibrium of the different conformations to the preferred configuration preselected for AR20.5 binding.

**Figure 5:**
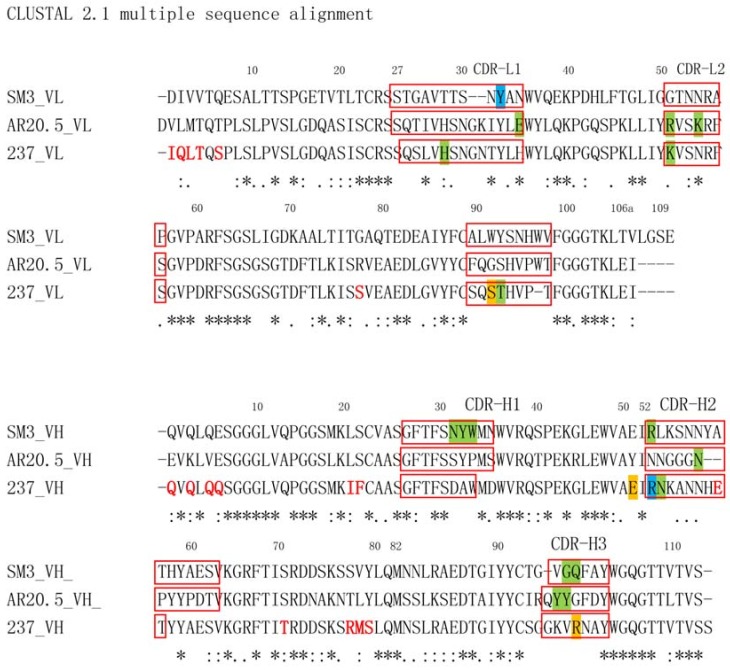
Sequence alignment of the scFV of anti-Muc1 antibodies and mAb237. The somatic mutations occurred at the framework region of mAb237 are highlighted as red bold letters. CDR loops of different antibodies are highlighted in red rectangles. Residues with green background interact with the peptide via hydrogen bonding and hydrophobic interactions, whereas residues highlighted in cyan background form interactions with both peptide and GalNAc. Residues only hydrogen bond with the carbohydrate moiety of the antigen are highlighted in yellow.

In summary, mAb237 wedges the sugar moiety in the defined binding groove and forms specific polar interactions that dictate the sugar species attached to the peptide, which implicates a unique glycosylation pattern occurring on the tumor-specific glycoprotein. Unlike mAb237, both AR20.5 and SM3 only have direct contacts with the peptide backbones and the sugar moieties are facing away from the peptide backbone binding pocket (Figure 5). The peptide moieties seem to contribute to the binding specificity in concerted with the sugar moieties. The preference of AR20.5 to the glycosylated epitope may be due to the differences in the immunogen used to produce the corresponding antibodies. Specifically, AR20.5 was generated using cancer-specific MUC1 glycoprotein as the immunogen, whereas that for SM3 is a “striped” MUC1 protein. The surface-binding property of the sugar may confer an evolutionary advantage for the host considering the heterogeneous nature of the glycosylation of tumor-specific MUC1 protein, where the antibodies can be selected and evolved faster in the maturation process. Lack of pronounced interactions between the sugar moieties and antibody combining sites indicates that sugar moieties play a different role to determine the specificity of the glycoprotein, i.e., not through defined polar and nonpolar interactions with the antibodies, rather through conjugation with certain MUC1 peptide sequences and induction of preferred binding configurations of the glycopeptides. Such implications await more structural elucidation of anti-MUC1 antibodies complexed with their cognate epitopes.

## 3. Hypothesized Functional Roles of Peptide and Sugar Portions: Four Types of Glycopeptide Signature Scanned by B Cells

Although the data are very few and limited, the above co-crystal structures of the antibody- glycopeptide give some clues about how a glycopeptide antigen stimulates the B cell receptor signaling, and triggers the differentiation and maturation of plasma cells to produce antibodies.

### 3.1. Direct Stimulation by Sugar (Glycans)

Cancer vaccines based on MUC1 glycans, with Theratope as the well-known example, have been extensively studied and tested in cancer patients. This is not surprising because several vaccines targeting microbial glycans are in clinical use, including carbohydrate capsules of Haemophilus influenzae type b, Streptococcus pneumoniae and Neisseria meningitides [60,61,62]. In contrast to microbial glycans, the immunogenicity of self-glycans are poor. A large-scale clinical trial of Theratope vaccine, which contains the clustered sialyl Tn antigens conjugated to KLH (Theratope), induced IgG antibodies with a median titer of 1:300 [63]. Immune tolerance to MUC1 self-glycans are hypothesized to be the major mechanism for poor immunogenicity of vaccines such as Theratope. This is supported by immunization data from knockout animals which are depleted of certain type of glycans. For example, in GM2 KO mice, IgG antibody titer to gangliosides can be induced up to 1:10,000, while no IgG antibody could be measured in immunized wild type mice [64].

### 3.2. Direct Stimulation of BCR by a Peptide Epitope

The peptide portion alone, even in the presence of a sugar modification, may induce antibodies which only react with the peptide structure. The binding of the antibody is not related to the sugar modification, and could not be inhibited by the free sugar. MUC1-specific antibodies of this class are exemplified by mAbs generated in patients vaccinated by non-glycosylated MUC1 peptides [34]. 

### 3.3. Glycan-Shield as Immune-Escape Mechanism to Suppress B Cell Stimulation by A Peptide

*N*-glycans and *O*-glycans of self-glycoproteins are known to be immune silent. Viruses use human *N*-glycosylation enzymes to modify their protein backbone and evade antibody recognition. The Env protein gp120 of HIV-1 virus is the most densely glycosylated protein in nature. On average, one of every 3 amino acids is *N*-glycosylated. Thus, the surface of exposed gp120 protein is an immune “silent” area.

To be an effective shield, the glycans must fit several criteria. First, the glycans must be present in the microenvironment during B cell development, so that the B cells exposed to these glycans can be deleted from the B cell pool. Secondly, such glycans must be large enough so that they can block the access of BCR to contact the peptide backbones. In view of the *O*-glycosylation of self-proteins, where the size of *O*-glycans are large enough, they may prevent the binding of antibodies to the protein backbone. This shield function of *O*-glycans is supported by the data that some MUC-1 specific antibodies do not recognize MUC1 molecules which carry core-2 elongated *O*-glycans, a glycan type normally present in normal tissues [65].

### 3.4. Glycan Modification to Improve the B Cell Stimulation by A Peptide

This improved binding to peptide portion may be achieved by two mechanisms. (1) The sugar does not bind to antibody directly, but alters the confirmation of a peptide, and increases the affinity of antibody binding; (2) The sugar portion has direct contacts with the antibody, and binding to the sugar portion improves the fitness of the peptide for binding.

Data in both mice and cancer patients suggest that glycan modification is the major mechanism for a glycopeptide to improve the B cell stimulation by the peptide portion. Sorensen et al. reported that the glycosylation of MUC1 peptide override immune tolerance [35]. Full glycosylation of all five *O*-glycosylation sites by Tn or STn sugars elicited strongest antibody response to MUC1 glycopeptides in mice. Further characterization of a mAb 5E5 generated by this vaccination approach indicated that GST(Tn)A motif is the antibody binding epitope [35]. Wandall et al. further demonstrated that the GSTA motif, only after glycosylation, is also recognized by serum from cancer patients vaccinated by a 106-mer MUC1 tandem repeat peptide with 25Tn *O*-glycans [66].

## 4. Glycopeptide Signature-Induced Monoclonal Antibodies: 3 Groups by Specificity

Glycopeptide-binding antibodies may be classified as three groups. Quantitative analysis of antibody specificity in these three groups (Table 1) are essential to evaluate the biological functions of glycopeptide-based vaccines.

### 4.1. Antibodies Which Only Bind to Peptide Portion

Most existing monoclonal antibodies only bind to the peptide backbone of MUC1 tandem repeats. In the past, three motifs of *O*-glycosylation were defined [48], and PDTR was found to be peptide backbone to be recognized by most mAbs generated. mAbs to the other two motifs (GVTS and GSTA) were rare.

### 4.2. Antibodies Which Only Bind to Sugar Portion

These mAbs include those that bind to abnormally expressed Tn and sialyl Tn glycans [67,68,69]. VL and VH sequences of monoclonal antibodies have been reported in both human and mouse species. In mice, the VH usages include IgHV1-78 germ-line VH sequence (Tn). The human, the VH usages include IGHV6-1 [69].

### 4.3. Antibodies Which Bind to Both Peptide and Sugar Portions

Examples of these mAbs are BW835, MY.1E12 (70), 115D8 [71] and KL-6 [72,73]. KL-6 mAb is a most characterized one whose epitope was identified by using a glycopeptide array-chip containing 60 glycopeptides. Matsushita found that the ST antigen attached to the PDTR sequence was the fine epitope for KL-6 antibody binding, while cross-reactivity to step-wise elongated tri-saccharide were observed. The Neu5Ac was found to be the most critical sugar for KL-6 binding. The K_d_ valueof KL-6 in binding to glycopeptides, which are modified by GalNAc, Galβ3GalNAc, Neu5Acα3Galβ3GalNAc, remains to be determined. 

## 5. Glycopeptide Signature-Guided Design of MUC1 Vaccine

### 5.1. The Use of Peptide Motif as Priming Immunogens

The role of peptide and glycan motifs in glycopeptide vaccine design have been extensively studied in the field of HIV glycopeptide vaccines. Synthetic peptides from the base of the V3 loop of gp120 was determined as germ-line-targeting immunogens to prime V3-glycan broadly neutralizing antibodies [73]. In contrast to the tip of the V3 loop, which only induce non-neutralizing antibodies, the base of the V3 loop that containing the GDIR motif could induce V3-glycan-specific broadly neutralizing mAbs. Affinity measurement found that the unmutated common ancestor (UCA) of the V3-glycan broadly neutralizing mAb DH270 did not bind to the N332 and N301 glycan-deleted gp140 but did bind to the aglycone V3 base peptide, suggesting that the GDIR motif activated a B cell clone which subsequently mutated to bind glycans. Induction of broadly neutralizing antibodies may require a sequential regimen of GDIR-motif followed by Env vaccination.

Several glycopeptide vaccines based on MUC1 tandem repeat sequences have been reported. The anti-tumor efficacy of such vaccines have been demonstrated in mouse models, and a dependence on antibody component have been proved. However, there is lack of immunobiological mechanisms for these vaccines. There are few monoclonal antibodies generated for these vaccines. The lack of data preclude further mechanistic studies. Presumably, if a MUC1 peptide motif, similar to the GDIR motif of the V3 [74], is designed as priming immunogen, it may be more effective than the intact glycopeptide motif to activate the glycopeptide-specific B cells, while the binding to glycans may be further induced when a glycopeptide is used to boost the immunization. 

### 5.2. Clustered Tandem Repeat Sequences for BCR Binding

We previously published that the K_d_ value for the binding of mAb 16A to MUC1 tandem repeat sequences significantly decrease when the tandem repeat numbers increase [27]. For sequences containing 3 tandem repeat units, the K_d_ is at 10 nM range. For sequences containing 5 tandem repeat units, the K_d_ reaches 1 nM range. These data suggest that long peptides containing 5 tandem repeat units can form optimal clusters of antigen-B cell receptor complexes with stronger affinity, and serve as better vaccine candidates. Several groups reported strong IgG responses induced by MUC1 TR glycopeptides conjugated to multi-functionalized hyper-branched polymers [75,76,77,78,79,80]. Using phage display technology, Huang lab recently showed that the density of Tn antigens displayed at phage particle is 5- to 10-fold higher than those displayed by cowpea mosaic virus capsid and other synthetic particles, and could induce antibody titer up to 1:250,000 [81,82]. The exact density of Tn epitopes displayed by phage surface could not be precisely measured, but is expected to be close to the Tn epitope displayed by natural occurring backbone of MUC1 tandem repeats.

### 5.3. The Choice of Peptide Backbone for Sugars

Previous studies suggest that PDTR motif is immune dominant in mice vaccinated by MUC1 proteins or peptides. In a multinational study on mAbs raised by natural MUC1 protein or synthetic long peptides containing multiple tandem repeating units, almost all monoclonal antibodies bind to PDTR backbone, either the backbone alone, or the backbone modified by Tn or other sugars [48]. In contrast, much fewer mAbs were found to bind to GSTA or GVTS motif. When Jurkat cell line transfected by a MUC1 gene was used to immunize mice, strong antibody reactivity was only found toward the PDTR motif (1:20,000 to 80,000). GSTA and GVTS motifs showed much weaker reactivity (1:640 to 2560). After extensive screening of multiple immunized mice, very few mAb clones could be generated against the GSTA and GVTS motifs ([37] and unpublished data).

PDTR motif is not the only epitope expressed by cancer cells. Immuno-histochemical staining and flow cytometry analysis using mAbs specific to GSTA motif (16A and 5E5) found that the GSTA epitope is abundantly expressed in almost all cancer cell lines tested ([27,36] and unpublished data). Interestingly, several mAbs specific to PDTR motif do not show broad recognition of tumor cells. These data suggest that cancer vaccines should not be limited to PDTR motif. GSTA and GVTS motifs should be targeted as well. 

If any of the PDTR, GSTA and GVTS motifs is immune dominant in vaccine development, sequential immunization regimen should be designed with immune-dominant epitopes administered following non-dominant epitopes.

### 5.4. Modification of Glycans on MUC1 Glycopeptides in Vaccine Design

MUC1-based tumor vaccine attracts great research interest during the past two decades. Many synthetic MUC1 peptide vaccines with various adjuvant designs were applied in clinical trials [83,84,85]. There have been extensive studies on the function of MUC1 peptide as T cell epitopes. Scheid et al. recently reported that the glycosylated MUC1-peptides, but not non-glycosylated MUC1 peptides, could stimulate T cells in prostate cancer patients [85]. This review focuses on the antibody induction by MUC1 glycopeptide vaccines, since there is no data to review on cloned T cell receptors binding to MUC1 glycopeptides in the last few decades.

The immunogenicity of carbohydrate moieties is usually lower than peptide backbones, therefore immunization of glycopeptides elicits anti-peptide antibodies in majority and much less anti-carbohydrate antibodies. To enhance immunogenicity of carbohydrate motif in MUC1 glycopeptide, unnatural sugar structures were applied in the vaccine design. Cipolla et al. reported a strategy using a neoglycopeptide containing a C-glycoside Tn mimic as the immunogen which could be efficiently presented and recognized by the T cell receptor [86]. Modification of amino acid in the Tn motif using unnatural alpha-methylserine analogue was also employed in Tn vaccine design [87]. Recently, a S-glycoside Tn neoglycopeptide with an optimal linker between the S-GalNAc and the amino acid indicated a helix-like conformation of the neopeptide for proper presentation of the sugar unit for anti-MUC1 antibody recognition [88]. Moreover, using unnatural-configuration of GalNAc instead of alpha-GalNAc in Tn MUC1 glycopeptide for immunization induced about 8-fold higher sera titers and induced the production of antibody against tumor cells [89]. Although these vaccines induced antibody responses toward MUC1 glycopeptide, there are no monoclonal antibodies have been generated in the process. Thus, there is no knowledge of the underlying mechanisms that antibody genes bind to MUC1 with modified unnatural Tn.

Besides the Tn mimics, MUC1 glycopeptide vaccines bearing unnatural TF and STn structures were also reported. Fluorine-substituted TF disaccharide analogue was employed in MUC1 glycopeptide vaccine design and induced very strong immune responses in mice and generated antibodies against the tumor-associated MUC1 in MCF-7 breast cancer cells [90]. Guo group reported a series of unnatural 5′-*N*-acyl STn derivatives which indicated improved immunogenicity and induced high titers of antigen-specific IgG antibodies [91,92,93]. Chen group developed an efficient chemoenzymatic method to synthesize a series of unnatural STn glycopeptide derivatives with modification on 5- and 9-position of the sialic acid moiety [18,94]. Recently, Ye et al. synthesized a number of unnatural *N*-modified S-linked STn glycoconjugates on a KLH carrier, that stimulated the production of antibodies against naturally occurring STn antigen in immunized mice [95]. These examples demonstrated a practical strategy using the unnatural MUC1 neoglycopeptides for vaccination with enhanced immunogenicity and presented a new concept for future MUC1-based vaccine design.

### 5.5. Double-Edged Sword: The Cross Reactivity of MUC1-Glycopeptide Binding Monoclonal Antibodies

**Figure 6:**
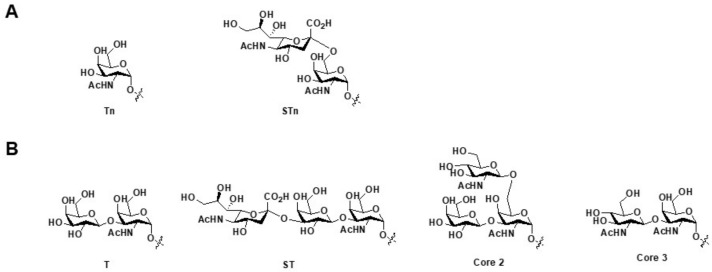
The structures linked to the cross reactivity of glyco-MUC1 antibody 5E5. (**A**) Sugar groups which can be recognized by 5E5; (**B**) Sugar groups which are not recognized by 5E5.

Cross reactivity of a MUC1-glycopeptide specific antibodies have been always a concern for their applications in diagnosis and therapy. The well-known 5E5 monoclonal antibody, for example, recognizes GST(Tn)A motif of the peptide. It also has cross reactivity to GST(STn)A glycopeptides. However, it does not have cross reactivity to GST(TF)A and GST(core3)A glycopeptides. These data suggest that 5E5 antibody bind to Tn, STn type of aberrantly glycosylated glycopeptides, but not core-1 or core-3 elongated glycopeptides [96], (Figure 6).

In other examples, mAbs such as BW835 and MY.1E12 were found to bind both core-3 and ST modified VT(modified)SA, but not Tn or STn modified VT(modified)SA [70]. SM3 was found to bind to unglycosylated PDTR motif and PDTR motif modified by Tn. It binds to lesser degree to PDTR modified by T and core-3 sugars, but does not bind to PDTR modified by ST sugar.

It is difficult to conclude whether the cross-reactivity of a glyco-MUC1 binding mAb plays a positive or negative role in their clinical applications. There are no general rules governing the cross reactivity of mAbs either. The selection criteria is that the mAb should be specific for tumor cells, but not healthy cells. Multiple ELISA-based clinical tests have been developed using mAbs with undefined glycopeptide specificity. For example, the 115D8 epitope is not clearly mapped in the DF3/115D8 test for CA153 in breast cancer patients [70].

## 6. Glycopeptide Signature-Guided Selection of Antibody Therapeutics

MUC1 glycoprotein is among the most promising targets for antibody-based drug development [97,98,99,100,101,102,103,104] (Table 2). Early efforts have been focused on IgG1 antibodies which block the MUC1 in the surface of cancer cells, according to the rational that MUC1 is an oncoprotein which protects the cancer cells against apoptosis because the intracellular domain of MUC1 binds to BAX protein [105]. Unfortunately, mAbs targeting MUC1 have failed to inhibit tumor growth in cancer patients. The mechanism may be due to the insufficient inhibition of anti-apoptotic pathway.

Antibody-dependent cell-mediated cytotoxicity is considered to be a major mechanism for antibody treatment of liquid tumors. For example, the efficacy of Rituximab (anti-CD20) is believed to be dependent on the phagocytic function of macrophages [106]. In vitro assays suggest that NK cells can exert MUC1 antibody-dependent cytotoxicity toward tumor cells [107,108], although the antitumor efficacy of ADCC has not been confirmed in vivo in cancer patients. 

In addition to recent efforts by monoclonal antibody drugs and inducing glyco-MUC1 binding antibodies with vaccines to treat cancer patients, other antibody-derived therapies are being actively explored. The unique requirement for the designs of such therapies are discussed as below.

### 6.1. Antibody-Drug Conjugate

In early years anti-MUC1 antibodies were conjugated to radioisotopes and used for both imaging and therapy of cancer. Such radiolabeled gained little success in improving patient survival in randomized clinical trials [100,101,102,103,104]. In general, most solid tumors are considered to be resistant to radiotherapy. Small molecule toxins are the main compounds currently being tested as candidates for being conjugated with antibodies for cancer therapy.

Antibodies conjugated to small molecule toxins have been approved to treat various types of cancer, including trastuzumab emtansine (targeting EGFR, [109]), brentuximab vedotin (targeting CD30, [110]), and gemtuzumab ozogamicin (targeting CD33, [111]). The ADC targeting a yet unknown glycopeptide epitope (CA6) of MUC1, SAR566658, has completed phase 1 trial in patients with CA6 positive advanced solid tumors [112]. Preclinical study of an ADC targeting sialy Tn (STn) glycan has been reported by Siamab Therapeutics [113].

The expression level of the targeted epitope determines not only the safety of antibody-drug conjugate, but also its efficacy. The therapeutic window is dependent on the ratio of target molecules on the surface of cancer cells to healthy cells. For example, it was determined that the cell surface expression of Her2 in breast cancer cell line is 10^6^ molecules per cell, while the healthy epithelial cells only express 10^4^ molecules per cell [114]. Studies on glyco-MUC1 epitopes in cancer versus healthy cells are currently ongoing.

Internalization is the other requirement for a mAb to be a good ADC candidate. Taking Her2 as an example, the internalization rate is a key criterion for selection of T-DM1 [115]. This allows the release of DM1 in the lysosome which permeates the lysosome membrane into the cytosol. In this regards, several anti-MUC1 antibodies exhibited excellent internalization while binding to the antigens on the cancer cell surface, which represent promising repertoire of antibodies to be developed as ADC candidates [34,112,113].

### 6.2. Bi-Specific Antibodies

The hypothesis that cancer-specific antibody can be fused with anti-CD3 antibody to redirect T cell mediated killing effect in cancer patients have been supported by the approval of catumaxomab (anti-EpCAM and anti-CD3, [116]) and blinatumomab (anti-CD19 and anti-CD3, [117]). Affinity to targets on cancer cell surface is the key for successful design of bispecific antibodies. The bispecific antibody only contains one Fab fragment which binds to cancer cells, while the other Fab fragment is from the anti-CD3 mAb. Thus, the affinity of Fab to cancer cell surface must be sufficient to bind tumor antigens.

There are very few data available for the K_d_ of MUC1-specific mAbs in glycopeptide binding. Wandall et al. reported a 1.5 nM of K_d_ for 5E5 antibody when binding to a 24-mer MUC1 glycopeptide containing one GST(Tn)A epitope [66]. Danielczyk et al. reported a PDT(Tn)R specific PankoMab with the affinity of nM range toward cancer cell lines, while the K_d_ toward PDT(Tn)R glycopeptide was not determined [118].

### 6.3. Chimeric Antigen Receptors 

Several MUC1 mAbs have been tested for CAR development. These include HFMG-2, SM3, and more recently 5E5 [36,119,120,121,122,123,124,125,126,127]. Safety is a concern for MUC1 mAb derived CARs, because MUC1 is a self-protein expressed by most healthy individual cells, especially in the lung. Thus, the abnormal glycosylation of MUC1 at cell surface is the key difference between cancer and healthy cells. Posey reported that 5E5 mAb and its single chain fragment could bind to cytosol of healthy lung epithelial and pancreatic duct cells, presumably Golgi complex where the MUC1 is glycosylated [36,120,121]. However, the cell surface of healthy cells does not express the epitope recognized by 5E5. 

To avoid the persistent cytokine storm caused by viral-transduced CAR T cells, electroporated T cells which transiently express CAR have been designed and tested for anticancer efficacy. The electroporated T cells only survive for less than a week in patients, and can be quickly eliminated by the body after the CAR T cells exert their anticancer functions [128,129]. Two types of large electroporators, from MaxCyte and Miltenyi, have been released for the electroporation of up to 10^11^ cells.

Tumor infiltration is a major barrier for CAR T therapy in solid tumor. Recently, NK cells, NKT cells, γδ T cells, and NK cell lines have been studied for their application in CAR-based cell therapy [130,131,132,133,134,135,136]. Heczey et al. reported excellent infiltration of CAR-NKT cells into tumor graft as compared to conventional CAR-CD8 T cells [136].

## 7. New Tools for Decoding Glycopeptide Signatures in High Throughput

(1) Glycopeptide array: the specificity of mAbs is always a most difficult issue when a mAb is used as a diagnostic tool. Several groups have published glycopeptide arrays to address this issue. In a 106 glycopeptide chip array designed by Westerlind group [65], MUC1 tandem repeat sequence containing abnormal and normal *O*-glycans at five sites of glycosylation were printed in a chip format, and used to test the polyclonal antibodies induced by synthetic MUC1 glycopeptide vaccines. It is anticipated that such glycopeptide chip arrays may be produced at a custom service level, with the cost of $50,000 for 106 glycopeptides each at microgram level, for 100 slides.
(2) Next generation sequencing and single B cell sequencing. The development of next generation sequencing and single cell sequencing technologies [137,138] has made it possible to generate a complete picture of B cell repertoire after the individuals are immunized by MUC1 glycopeptide vaccine. By focusing on syndecan+ memory B cells and plasma cells, those mAb clones which expand after being stimulated by glycopeptide vaccines can be selected, and expressed as Fab at high throughput, allowing the functional studies of sequenced BCRs.
(3) The humanized antibody transgenic mice. Several humanized antibody mouse models have been developed which have the mouse IgH and IgL loci replaced by human ones, and humanized antibodies have been generated [139,140,141,142,143]. The other interesting tool is the human IL6 knock-in mice which allows better engrafting of human cord blood cells to develop into mature human T and B cells. Using OVA as an antigen, Flavell group reported high affinity antibody response and isolated humanized mAb clones in this model [144]. Antibodies generated from mice engrafted with human lymphocyte progenitors serve as a unique tool for answering difficult questions, such as which motif of MUC1 tandem repeat unit is the immune dominant epitope.

## 8. Outlook

### 8.1. Possible Breakthrough in Antibody Diagnosis in Next 5 Years 

MUC1 has been reported to be expressed by exosomes from both cancer cell lines and serum of cancer patients [145,146,147,148], although major technical challenges still exist for the practical application of using flow cytometry analysis to measure glycoprotein levels in exosomes. With the advancement of exosome technology, CTC technology, MUC1-specific antibodies may be used to enrich exosomes. This may be achieved by traditional enrichment of exosomes by antibody-conjugated beads, or more advanced technologies such as microfluid systems [149,150,151,152].

MUC1 glycopeptide array may be used as the tool to evaluate the patients’ antibody response to cancer. The combined use of MUC1 glycopeptides with other tumor antigens currently in clinical tests, might further increase the sensitivity and specificity for early cancer detection [153,154].

### 8.2. Possible Breakthrough in Antibody Therapy in Next 5 Years

Phase 2 and phase 3 clinicals for MUC1 antibody-derived therapeutics will be completed in clinics, such as antibody-drug conjugate and CAR T and CAR NK therapies targeting MUC1 positive solid tumors.

### 8.3. Multi-Valent Glycopeptide Vaccine R&D in Next 5 Years

MUC1 vaccines which induce antibodies to all the 3 regions of *O*-glycosylation will be developed. Vaccines which induce high titer of broadly-neutralization antibodies will be developed, i.e., antibodies which cross-react to all glycoforms decorating a same site of *O*-glycosylation and peptide backbone. Some of these vaccines may enter clinical trials. The efficacy of these vaccines will be improved by combination with recently approved immune therapeutics such as PD1-blockade antibodies. Furthermore, the mechanism of MUC1 glycopeptides as T cell epitopes will be better understood. MUC1-glycopeptide-specific T cell receptors may be cloned which will lead to studies on co-crystal structures of glycopeptides complexed with MHC Class I molecules and T cell receptors. The long-proposed hypothesis that MUC1 peptides serve as noncanonical ligands for MHC Class I may be examined in cancer patients [85,155,156,157,158,159,160,161,162,163,164,165,166,167].

## Figures and Tables

**Table 1:** MUC1 Glycopeptide-induced antibodies.

Antibody Binding Site	Examples	Epitopes	VH Usage	References
Peptide backbone	DF3 C595 GP1.4	TRPAPGS RPAP DTRP	Diverse	[48] [48] [48]
Sugar	B72.3 4E10, 4G2	clustered sialyl-Tn three consecutive Tn	IgHV1-78 (mouse) IGHV6-1(human)	[67,68] [69]
Both peptide backbone and sugar	BW835 MY.1E12 115D8 KL-6 5E5	GVT(Galβ1,3GalNAc)SA GVT(Galβ1,3GalNAcS) Sialyl-Tn MUC1 tandem repeat PDT(Neu5Ac α2,3Galβ1,3GalNAc)RPAP GST(Tn)A	Diverse	[70] [70] [71] [72,73] [35]

**Table 2:** Clinical trials of MUC1-specific monoclonal antibodies.

Year Published	Clone/Product Name	Epitope	Active Ingredient	Efficacy	PI and Reference
2017	SAR566658(CA6)	Glycosylated PDTR	Humanized mouse mAb conjugated to maytansinoid	Phase 1 trial; safe; encouraging antitumor activity; selected for further clinical development	Sanofi, [112]
	CAR TAG72	STn sugar	CAR T cells	Safe; trafficking of CAR T cells to tumor.	Hege, [127]
	CAR SM3	Glycosylated PDTR	CAR T cells	Remission of tumor (intratumoral injection)	Yang, [123]
2016	PankoMab-GEX	Glycosylated PDTR	Humanized mouse mAb	Phase 1 trial; safe, promising efficacy in advanced diseases	Fiedler, [98]
2011	AS1402(AR20.5)	Glycosylated PDTR	Humanized mouse mAb	Phase 2 trial; no improvement in clinical outcome	Ibrahim, [97]
2006	R1549(HMFG1)	Glycosylated PDTR	Mouse mAb conjugated with ^90^Y-l	Phase 3 trial; no improvement in clinical outcome	Verheijen, [99]
2000	C595-^67^Cu	Glycosylated PDTR	Mouse mAb conjugated with ^67^Cu	Phase 1 trial; initial tumor uptake was high after intravesical injection	Hughes, [100]
	CMB401	Unknown MUC1 sequence	Humanized mouse mAb conjugated with calicheamicin	Phase 1 trial; safe; reduction in CA125 and tumor bulk observed in some patients	Gillespie, [101]
1998	Bre-3	Unknown MUC1 sequence	Humanized mouse mAb conjugated with ^111^In and ^90^Y-l	Phase 1 trial; excellent tumor localization, good tumor dosimetry, and low immunogenicity	Kramer, [102]
1995	111In-NCRC48	Unknown MUC1 sequence	Mouse mAb conjugated with ^111^In and ^90^Y-l	Phase 1 trial; uptake by tumor after intravesical injection to treat bladder cancer	Kunkler, [103]
1994	Bre-3	Unknown MUC1 sequence	Mouse mAb conjugated with radioisotopes	Phase 1 trial; rapid clearance of antibody due to immunogenicity of mouse mAb	Kramer, [104]

## Abbreviations

The following abbreviations are used in this manuscript:

BCR	B cell receptor
CAR	chimeric antigen receptor
CDR	complementarity-determining region
CTC	circulating tumor cell
DM1	Mertansine
FR	framework region
IgG	immunoglobulin G
IgH	immunoglobulin heavy chain
IgL	immunoglobulin light chain
KLH	keyhole limpet hemocyanin
mAb	monoclonal antibody
NK cells	natural killer cells
NKT cells	natural killer T cells
OVA	Ovalbumin
ST antigen	Neu5Ac α3 Gal β3 GalNAc α-Ser/Thr
STn antigen	Neu5Ac α6 GalNAcα-Ser/Thr
T antigen	Gal β3 GalNAc α-Ser/Thr
Tn antigen	GalNAc α-Ser/Thr
TR	tandem repeat
VH	heavy chain
VL	light chain
